# A Two-Dimensional Human Minilung System (Model) for Respiratory Syncytial Virus Infections

**DOI:** 10.3390/v9120379

**Published:** 2017-12-10

**Authors:** Esmeralda Magro-Lopez, Trinidad Guijarro, Isidoro Martinez, Maria Martin-Vicente, Isabel Liste, Alberto Zambrano

**Affiliations:** 1 Functional Unit for Research into Chronic Diseases (UFIEC), Institute of Health Carlos III, 28220 Madrid, Spain; emagro@externos.isciii.es (E.M.-L.); tguijarro@externos.isciii.es (T.G.); iliste@isciii.es (I.L.); 2 Spanish National Center for Microbiology (CNM), Institute of Health Carlos III, 28220 Madrid, Spain; imago@isciii.es (I.M.); maria.martinv@externos.isciii.es (M.M.-V.)

**Keywords:** human embryonic stem cells, epithelial cells, respiratory syncytial virus, minilung

## Abstract

Human respiratory syncytial virus (HRSV) is a major cause of serious pediatric respiratory diseases that lacks effective vaccine or specific therapeutics. Although our understanding about HRSV biology has dramatically increased during the last decades, the need for adequate models of HRSV infection is compelling. We have generated a two-dimensional minilung from human embryonic stem cells (hESCs). The differentiation protocol yielded at least six types of lung and airway cells, although it is biased toward the generation of distal cells. We show evidence of HRSV replication in lung cells, and the induction of innate and proinflammatory responses, thus supporting its use as a model for the study of HRSV–host interactions.

Human respiratory syncytial virus (HRSV) is the leading viral agent causing severe pediatric respiratory disease worldwide, and is associated with significant morbidity and mortality [1,2]. HRSV causes a descending infection, commencing in the upper respiratory tract and progressing to the lower respiratory tract. In the conducting airways, HRSV infection is restricted to the superficial layer (apical ciliated cells) and, occasionally, non-ciliated cells [3,4,5,6]. Most in vitro studies on HRSV–host interaction were undertaken in either immortalized airway-derived cell lines, such as HEp-2 and A549, or primary epithelial cells derived from human lung airways. It was then followed by an outflow of a series of Transwell systems of well-differentiated primary airway epithelial cell cultures (WD-PAECs) displaying a pseudostratified epithelium comprising basal, globet, and ciliated cells [4,5,6,7]. These cultures mimic the in vivo airway epithelium regarding cilia coverage and beating, and mucus production. An additional advantage is that the HRSV–epithelium interaction can be studied in the absence of immune cells. Two major disadvantages of these models are the low availability of precious clinical material, and the narrow cell spectrum obtained. Current alternative in vitro strategies would imply the use of pluripotent stem cells (PSCs) for the generation of human minilungs that mimic the natural lung and offer an immediate and indefinite availability. The ability of hPSCs to differentiate into airway and lung epithelial cells have multiple applications, including autologous transplantation, study of lung development, drug screening, and modeling of respiratory diseases [8]. Snoeck’s group has recently described efficient strategies to differentiate hPSCs into lung and airway epithelial cells (two-dimensional (2D) minilung) and to generate a 3D lung bud organoid [9,10,11]. Among the embryonic stem cell (ESC) lines tested, they focused on RUES (US National Institutes of Health) that was more efficient for anterior foregut endoderm (AFE) induction. One of the initial applications of this lung bud organoid included the infection by HRSV. The authors observed the swelling, detachment, and shedding of infected cells into the lumen of the branching structures.

In order to set up an in vitro study model for HRSV infection based on PSCs, we have generated a two-dimensional minilung from a hESC line. Essentially, we have followed the same strategy described by Snoeck’s group in their initial works [10,11]. Briefly, the reported sequence of differentiation to epithelial cells, and the relevant features of their strategy are summarized in Table 1. Some of our modifications on that strategy are related to:

(i) Standard methods of expansion and maintenance of hESCs
(ii) Use of NOG (NOGGIN) instead of dorsomorphin
(iii) Cell density in replatings
(iv) Sizes of multiwell plates used
(v) Final assembly of the minilungs on glass chamber slides
(vi) Addition of primary lung fibroblasts to the generated epithelial cells

The complete description of our strategy and protocols is available online as Supplementary Information. The hESC cell line used in our study was AND-1 (ANDalucia-1) from the Spanish National Stem Cell Bank (Granada, Spain). AND-1 was established from the inner cell mass of a day 5 human blastocyst. Figure 1A shows the typical morphology of an AND-1 colony growing along with feeder cells (mouse embryo fibroblasts (MEFs)). When maintained in an undifferentiated state, AND-1 shows a strong and homogenous expression of SOX2 (SRY (Sex Determining Region Y)-box 2), a marker of pluripotency (center and right panels, Figure 1A). To maximize the levels of lung specification, the activity of key pathways in endoderm induction and lung development, such as activin-A, FGFR (fibroblast growth factor receptor), BMP (bone morphogenic protein), TGF-β (Tumor Growth Factor β), or WNT (Wingless-Related Integration Site), were precisely switched on/off at specific times by the action of specific agonists and inhibitors (Table 1). Representative micrographs at different checkpoints of the sequential differentiation are shown in Figure 1B. These checkpoints are the formation of definitive endoderm in suspension (embryoid bodies, EBs), the induction of anterior definitive endoderm (AFE), and the induction of lung progenitors at day 21. To verify the presence of lung specification we evaluated by qRT-PCR the expression of *FOXA2* (Forkhead Box A2) and *NKX2-1* (NK2 Homeobox 1), key transcription factors involved in lung development and homeostasis. Around day 21, the expression of lung progenitor markers should be already evident when applying this differentiation protocol. As shown in Figure 1C, both transcription factors were expressed at days 21 and 29. The robust and homogeneous expression of NKX2-1 was confirmed by indirect immunofluorescence, thus unequivocally demonstrating the efficient expression of lung field specification in virtually the entire cell population (Figure 1D). Terminal differentiation of progenitors required some additional weeks of treatment with the lung and airway maturation cocktail (Table 1 and supplementary information). As reported before [10], we confirmed the expression of markers of terminal differentiation around day 50 (data not shown) [12] a time from which the amount of mature epithelial cells gradually increases. Continuous incubation with the maturation cocktail gave rise to cultures with an increasing heterogeneity. Microscopical examination of these cultures showed the presence of flat cells with a crescent shape morphology, and of granular and roughly cuboidal-shaped cells, likely corresponding to alveolar cells type I (ATI) and type II (ATII), respectively (Figure 1E). We performed analysis of lung and airway cell markers by qRT-PCR at different time points. At day 106, all tested markers of mature airway and lung epithelial cells were detected. These included *AQP5* (Aquaporin 5) and *PDPN* (Podoplanin) [ATI cells], *SFTPA*, *B*, *C*, and *D* (Surfactant Protein A-D; ATII cells), *TP63* (Tumor Protein p63; basal cells), *MUCIN5AC* (Mucin 5AC; globet cells), *SCGB1A1* [Secretoglobin Family 1A Member 1 or *CC10* (Clara cell 10-KDa protein); Clara cells] and *FOXJ1* (Forkhead Box J1; ciliated cells) (Figure 1F). As expected, the expression of these markers was negligible, or absent, at day 21, when the cell population is predominantly composed of lung field progenitors (Figure 1G). As previously described [10,11], this protocol of differentiation yielded cultures enriched in alveolar cells. Once we confirmed the efficiency of differentiation under hypoxic conditions, the cultures were switched to normoxic conditions, to mimic the normal physiological conditions. In addition, we incubated the cultures with maturation medium without dexamethasone for at least two days, to avoid the anti-inflammatory effects induced by dexamethasone. To emulate the cellular composition of the lung, the mature epithelial cells generated were cocultured with primary human lung fibroblasts on fibronectin-coated glass chamber slides (Supplementary Information). This enriched version of the minilung was infected with the Long strain of HRSV at two multiplicities of infection (moi), 1 and 0.1. After virus adsorption, the inocula were removed, and the cultures were incubated for additional 72 h for the infections at moi 1, or 48 h in the case of moi 0.1. Infected cultures at moi 1 showed clear signs of infection (Figure 2A). Virus titers of the clarified supernatants were 4 ± 0.2 × 10^6^ pfu (plaque forming units) per mL, indicative of HRSV replication in these cultures. We also detected the expression of ATI and ATII markers in some cells that remained attached to the substrate (Figure 2B). In addition, the coexpression of mature surfactant protein C (SFTPC) and viral antigens was detected in some individual cells (cells labeled as “a” and “b” in pictures from Figure 2C). Infections at lower moi allowed the formation of the typical syncytia as those observed during the propagation of HRSV in HEp-2 or A549 cell lines (Figure 3A). Virus titers of the clarified supernatants (4.25 ± 0.25 × 10^4^ pfu/mL) were significantly lower to those of cultures infected at moi 1. The coexpression of alveolar markers (PDPN or SFTPC) and viral antigens was detected in some individual cells (left and center panels, Figure 3B). Alpha smooth muscle actin (α-SMA or ACTA2) served to detect the presence of the myofibroblasts cocultured with the epithelial cells. As shown in Figure 3B (right panel), actin fibers were observed in the cytoplasm of the majority of the myofibroblasts. In addition, low levels of viral antigen expression were also observed in some myofibroblasts (cells “c” and “d” of Figure 3B, right panel), indicative of HRSV replication. Typically, HRSV infection of the airway epithelium induces an innate immune response leading to the secretion of cytokines and chemokines by the epithelium. CXCL8 (C-X-C Motif Chemokine Ligand 8), CXCL10, IL6 (Interleukin 6), IL1B (Interleukin 1 β), TNF (Tumor Necrosis Factor, TNF α), etc., are among the chemokines and cytokines increased in bronchoalveolar lavages, nasal aspirates, and blood of HRSV-infected infants [1]. To determine whether the infection of minilungs by HRSV reflects the in vivo innate and proinflammatory immune responses to HRSV, we performed qRT-PCR analysis of HRSV- and mock-infected minilungs. Consistent with the HRSV-induced gene expression in vivo or in WD-PAECs [1,5], *IL6*, *CXCL8*, and *TNF* expression were increased in HRSV-infected minilungs (Figure 3C). Moreover, the expression of *DDX58* (DExd/H-Box Helicase 58) receptor, that triggers innate immunity and inflammation and of *ISG15* (ISG15 Ubiquitin-Like Modifier)*,* an innate immune protein with antiviral activity, were also increased in HRSV-infected minilungs, consistent with previous reports [13,14,15,16] (Figure 3C).

Taken together, the data presented here confirm that the generated 2D-minilung is an adequate model for the study of HRSV–host interactions. HRSV tropism in humans includes mainly ciliated and alveolar epithelial cells [17,18], thus making this minilung a convenient study model. The differentiation protocol is biased toward the generation of distal cells as reported by Huang et al. [10,11]. It has been proposed that CX3CR1 (C-X3-C Motif Chemokine Receptor 1) expressed by ciliated cells is an important surface molecule for HRSV infection [19,20]. This fact precludes the use of these 2D minilungs to study the contribution of CX3CR1-mediated HRSV entry to cells. These minilungs display, however, a 2D array of cells in which viruses may have an equal access to all the epithelial cell types. Moreover, two-dimensional minilungs can be easily enriched by adding fibroblasts or endothelial cells, and serve as the basic cellular assembly to generate three-dimensional structures on hydrogels (sodium alginate microspheres, for instance) or poly (lactic acid) (PLA)/poly (d,l-lactic-coglycolic acid) (PLGA) biodegradable scaffolds. Finally, the proper exploitation of lung organoid versions recently described [9,21] by using stem cell technologies, especially patient-specific, is a great promising approach for individual medical therapies.

## Figures and Tables

**Figure 1:**
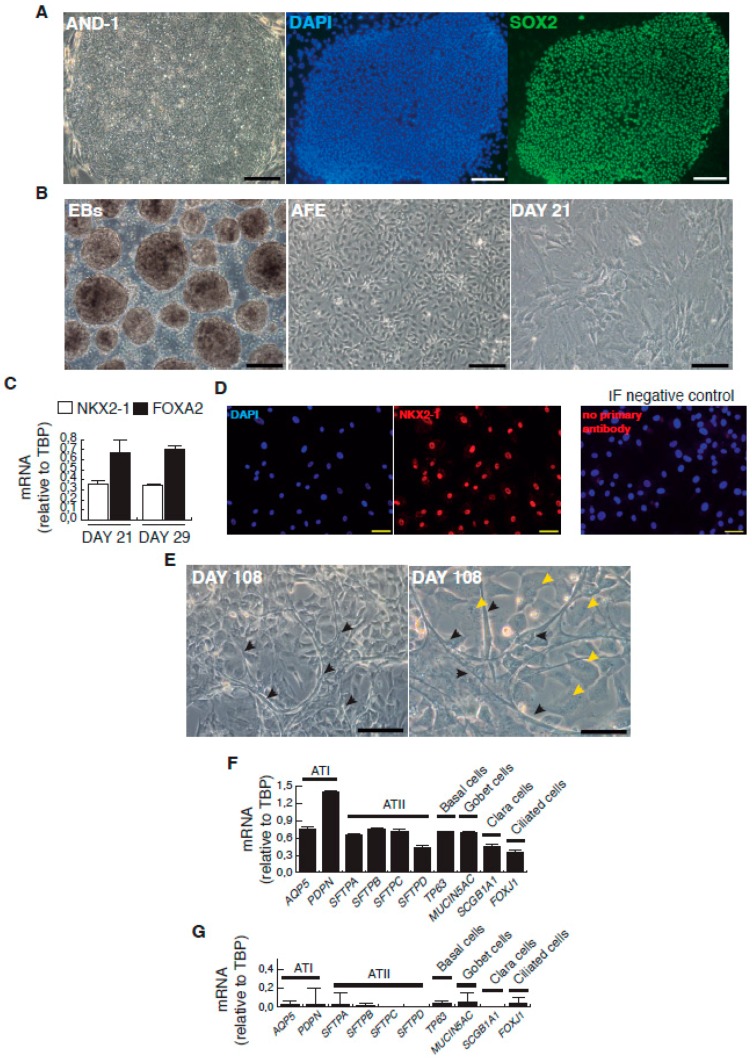
Representative micrographs of the sequential cell differentiation and expression markers. (**A**) AND-1 (ANDalucia-1) colony growing along with feeder cells (left panel; scale bar: 200 μm); expression of SOX2 (SRY (Sex Determining Region Y)-box 2) in an undifferentiated colony of AND-1 (center and right panels; scale bar: 100 μm); (**B**) Representative micrographs at different checkpoints. EBs (embryoid bodies), AFE (anterior foregut endoderm) and day 21 of differentiation; scale bar: 100 μm; (**C**) Levels of expression of *NKX2-1* (NK2 Homeobox 1) and *FOXA2* (Forkhead Box A2) at days 21 and 29 of differentiation; (**D**) Detection of the expression of *NKX2-1* by indirect immunofluorescence at day 29; scale bar: 100 μm. Immunofluorescence (IF) control: no primary antibody, on the right panel; (**E**) Representative micrographs of the minilung at day 108 of differentiation. Black arrowheads signal cells with a typical flat and crescent shape morphology denoting alveolar type I cells (ATI cells); yellow arrowheads signal cells with ATII morphology. Scale bars: 100 μm and 50 μm; (**F**) Levels of expression [relative to TBP (TATA Box Binding Protein)] of lung and airway epithelial cells markers; (**G**) Levels of expression (relative to TBP) of lung and airway epithelial cells markers at an early stage of differentiation (day 21).

**Figure 2:**
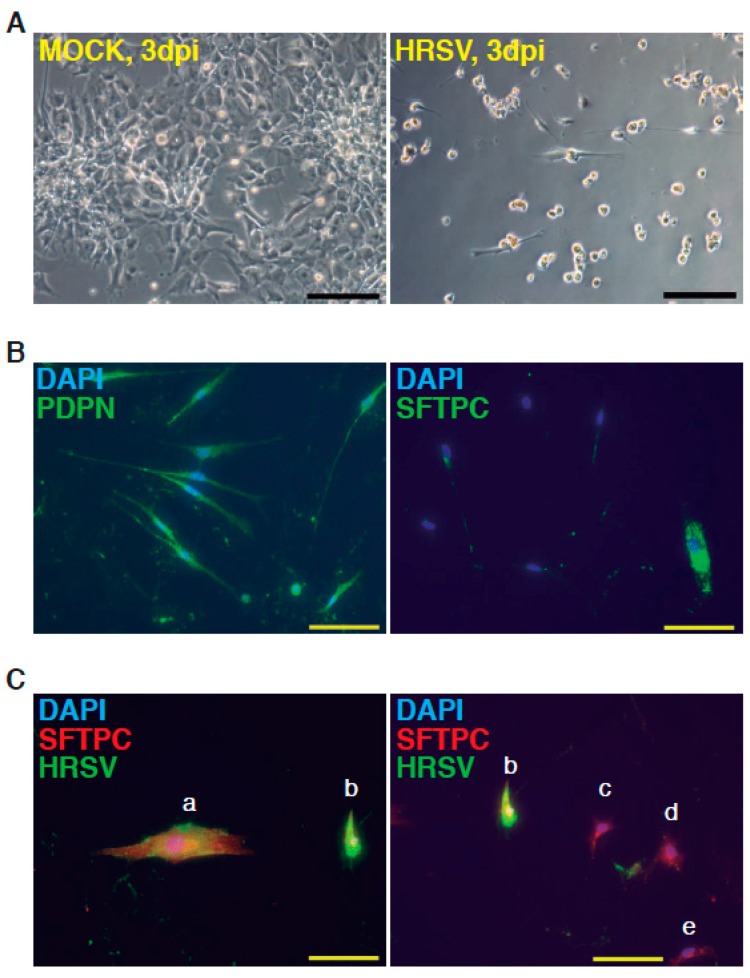
Infection of minilungs by human respiratory syncytial virus (HRSV) [moi (multiplicity of infection) 1]. (**A**) Representative bright field micrographs at 3 dpi (days post infection); scale bar: 100 μm; (**B**) Detection by indirect immunofluorescence of ATI cells marker podoplanin (PDNP) and of ATII marker (SFTPC; mature surfactant C protein) in HRSV-infected cells; scale bars: 50 μm; (**C**) Double detection of SFTPC and viral antigens by indirect immunofluorescence. Cells labeled as “a” and “b” show the merged colors. Cells “c”–”e” only express SFTPC. HRSV antibody used: mix of mAbs directed against HRSV (Supplementary Information).

**Figure 3:**
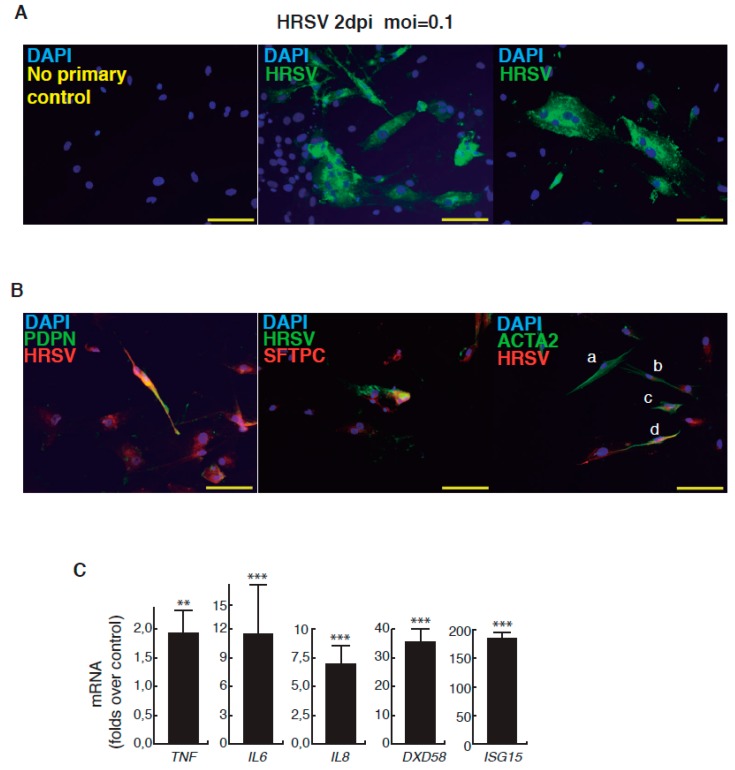
Infection of minilungs by HRSV (moi 0.1). (**A**) Detection by indirect immunofluorescence of viral antigens by using a mix of mAbs directed against HRSV (Supplementary Information); 2 dpi: 2 days post-infection; scale bars: 50 μm; (**B**) Left panel: double detection of podoplanin (PDPN), a marker of ATI cells, and of viral antigens (F glycoprotein); HRSV antibody used is a rabbit polyclonal antibody raised against the F glycoprotein (supplementary information). Center panel: double detection of mature surfactant C protein (SFTPC), a marker of ATII cells, and of viral antigens. HRSV antibodies used: mix of mouse mAbs directed against HRSV. Right panel: double detection of ACTA2 (α-SMA (alpha smooth muscle actin), marker of myofibroblasts) and of viral antigens. Cells “a” and “b” show no significant reactivity to HRSV antibodies; cells “c” and “d” show the merged color indicative of a moderate infection. HRSV antibodies used: mix of mouse mAbs directed against HRSV; scale bars: 50 μm; (**C**) Relative levels of expression of representative genes of the proinflammatory and innate immune responses induced by HRSV. Infections were performed at moi 0.1 and total RNA was extracted at 2 dpi. Values are folds over control (uninfected minilungs). (**, *p* <0.01; and ***, *p* < 0.001.)

**Table 1:** Chronogram of the differentiation strategy followed to generate the two-dimensional minilung from hESCs (human embryonic stem cells).

Day	Process	Atmosphere/Observations	Factors, Inhibitors and Hormones Used	Targeted Patwways Activation Inhibition
	Matrigel depletion of MEFs	95% air/5% CO_2_		
0	Primitive streak induction	5% O_2_/95% N_2_/5% CO_2_ Low attachment plates	Wnt3a, BMP4, ROCK inhibitor	WNTs BMP apoptosis
1–3	Definitive endoderm induction	5% O_2_/95% N_2_/5% CO_2_ Low attachment plates	Activin A, BMP4, hbFGF, ROCK inhibitor	ACVR BMP FGFR apoptosis
4–6	Anterior foregut induction	5% O_2_/95% N_2_/5% CO_2_ fibronectin coated-plants	Dorsomorphin or NOGGIN, SB431542, IWP2	BMP TGF-β WNTs
6–15	Lung progenitor induction and expansion	5% O_2_/95% N_2_/5% CO_2_ (intermediate incubation under 95% air/5% CO_2_) fibronectin coated-plates	CHIR99021, KGF, FGF10, BMP4, EGF, all-trans retinoic acid	Wnts BMP FGFR2b (Ventralization)
16–25	Lung progenitor induction and expansion	5% O_2_/95% N_2_/5% CO_2_ fibronectin coated-plates	CHIR99021, KGF, FGF10	Wnts FGFR2b
26–~1 year	Lung and airway epithelial maturation	5% O_2_/95% N_2_/5% CO_2_ fibronectin coated-plates	CHIR99021, KGF, FGF10, Dexamethasone, IBMX, cAMP	Wnts FGFR2b (Alveolar maturation)

hESCs: human embryonic stem cells; MEFs: mouse embryonic fibroblasts; BMP4: bone morphogenic protein 4; ROCK: rho-associated coiled-coil containing protein kinase; ACVR: activin A receptor; FGFR: fibroblast growth factor receptor; TGF: tumor growth factor; IWP2: WNT inhibitor; CHIR99021: WNT activator; KGF: keratinocyte growth factor; FGF: fibroblast growth factor; EGF: epithelial growth factor; IBMX: 3-isobutyl-1-methylxanthine; cAMP: 3′,5′ cyclic adenosine monophosphate

## Supplementary Materials

Supplementary materials can be found at www.mdpi.com/1999-4915/9/12/379/s1.

## Abbreviations

*ACTA2*	Alpha Smooth Muscle Actin (α-SMA)
AFE	Anterior Foregut Endoderm
*AQP5*	Aquaporin 5
ATI	Alveolar Type I Cells
ATII	Alveolar Type II Cells
BMP	Bone Morphogenic Protein
*CXCL10*	C-X-C Motif Chemokine Ligand 10
*CXCL8*	C-X-C Motif Chemokine Ligand 8 (IL8)
CX3CR1	C-X3-C Motif Chemokine Receptor 1
DE	Definitive Endoderm
dpi	Days Post-Infection
*DXD58*	DExd/H-Box Helicase 58 (RIG-1: Retinoic Acid Inducible Gene 1)
EBs	Embryoid Bodies
*FGF*	Fibroblast Growth Factor
*FGFR*	Fibroblast Growth Factor Receptor
*FOXA2*	Forkhead Box A2
*FOXJ1*	Forkhead Box J1
hESC	Human Embryonic Stem Cells
HRSV	Human Respiratory Syncytial Virus
*IL1B* *IL6*	Interleukin 1 β Interleukin 6
*ISG15*	ISG15 Ubiquitin-Like Modifier
mAb	Monoclonal Antibody
MEFs	Mouse Embryonic Fibroblasts
moi	Multiplicity of Infection
*MUCIN5AC*	Mucin 5AC, Oligomeric Mucus/Gel-Forming
*NKX2-1*	NK2 Homeobox 1
*NOG*	Noggin
*PDPN*	Podoplanin
pfu	Plaque Forming Unit
PSC	Pluripotent Stem Cells
qRT-PCR	Quantitative Real-Time RT-PCR (Reverse Transcription Polymerase Chain Reaction)
*SCGB1A1*	Secretoglobin Family 1A Member 1; *CC10*
*SFTPA*	Surfactant Protein A
*SFTPB*	Surfactant Protein B
*SFTPC*	Surfactant Protein C
*SFTPD*	Surfactant Protein D
*SOX2*	SRY (Sex Determining Region Y)-box 2
*TBP*	TATA Box Binding Protein
*TGFB1*	Tumor Growth Factor β
*TNF*	Tumor Necrosis Factor (TNF α)
*TP63*	Tumor Protein p63
WNT	Wingless-Related Integration Site
μm	Micrometers
