# Supplementary material for: A Two-Dimensional Human Minilung System (Model) for Respiratory Syncytial Virus Infections
Source: Viruses. 2017 Dec 10;9(12):379. doi: 10.3390/v9120379 (PMC5744153; doi:10.3390/v9120379)
Supplement: Supplementary file 1 [file viruses-09-00379-s001.pdf]

Supplementary information

Online materials and methods

Maintenance of human embryonic stem cells (hESCs)

AND-1, a human embryonic stem cell line was obtained from the "Biobanco de células madre de Granada" (ISCIII, Spain); passages 27-40. Mouse embryonic fibroblasts (MEFs) were obtained at 13.5 days *post-coitum* from C57BL/6 mice as described previously[1]. MEFs were mitotically inactivated by an overnight treatment with 2 μg/mL of mitomycin C (cat.#M4287; Sigma-Aldrich) and plated at a density of approximately 16000 cells/cm^2. AND-1 cells were cultured on MEFs under standard conditions (http://stembook.org). The maintenance medium was composed of KO-DMEM (cat.#10829-018 Gibco; Life Technologies), 20% KO serum replacement (cat.#10828-010 Gibco; Life Technologies), 0,1 mM β-mercaptoethanol (cat.# 21985-023 Gibco; Life Technologies), 2 mM Glutamax (cat.#35050-061, Gibco; Life Technologies), non-essential aminoacids (cat.#11140-050 Gibco; Life Technologies), primocin (cat.#12I05-MM, InvivoGen). The medium was filtered by using 0,22-μ pore filter systems (cat.#431097, Corning) and 10 ng/mL recombinant human basic Fibroblast Growth Factor (hbFGF) (cat.# PHG6015, Invitrogen) and 10 μM Y-27632 (cat.#1254, Tocris R&D Systems) were added before use. Medium was changed daily and cells were passaged either by enzymatic (collagenase IV method) (collagenase IV: cat.#11140-050, Gibco; Life Technologies) or mechanical procedures (http://stembook.org). Cells were maintained in an undifferentiated state in a 5% CO_2/air environment. The differentiation process was carried-out in a 5% CO_2/5% O_2/95% N_2 environment (Galaxy 48R incubator (New Brunswick)) unless otherwise indicated.

Primitive streak formation and induction of definitive endoderm (DE)

Primitive streak formation (24h; day 0) and endoderm induction (days 1 to 4) were performed in serum-free differentiation (SFD) *medium*. SFD was composed of a mix of IMDM:F12 (3:1) *media* (cats.#B12-722F and 10-080 CVR, Corning), supplemented with N2 (cat.#17502-048, Gibco; Life Technologies), B27 (cat.#17504-044, Gibco; Life Technologies), 2 mM Glutamax (cat.#35050-061 Gibco; Life Technologies), 1% penicillin-Streptomycin (DE17-602E, Lonza), 0,05% bovine *serum* albumin (cat.#A7906, Sigma-Aldrich). The medium was filtered by using 0,22μ-pore filter systems (cat.#431097, Corning) and 50 μg/mL of ascorbic acid (cat.#A4554, Sigma-Aldrich) and 0,04 μl/mL of monothioglicerol (cat.#M6145, Sigma-Aldrich) were added before use. AND-1 were plated on Matrigel-coated (cat.#354230, Life Technologies) plates for 48h to deplete residual MEFs. Cells were then briefly trypsinized into small 3-10 cell clumps and the reaction was halted with stop *medium* [IMDM medium (BE12-722F) supplemented with 50% fetal bovine *serum* (F7524, Sigma-Aldrich), 2 mM Glutamax, 1% penicillin-streptomycin and 30 ng/mL DNase I (cat.#260913-10MU, Calbiochem)]. Cells were then centrifuged 5min at 850 rpm and washed carefully two times with an excess of SFD *medium*. To form embryoid bodies (EBs), the clumps were plated onto low-attachment 6-well plates (cat.#3471, Corning) in SFD *medium*. For primitive streak formation, 10 μM Y-27632, 10 ng/mL Wnt3a (cat.#5036-WN, R&D Systems) and 3 ng/mL of human BMP4 (cat.#314-BP, R&D Systems) were added to SFD *medium*. EBs were then collected, resuspended carefully in endoderm induction

medium containing 10 μM Y-27632, 0,5 ng/mL human BMP4, 2,5 ng/mL hbFGF and 100 ng/mL of human Activin (cat.# 338-AC, R&D Systems). Cells were fed after 36-48h, depending on cell density, by removing half the old *medium* and adding half fresh *medium*.

Induction of anterior foregut endoderm (AFE)

On day 4,5 or 5, EBs were dissociated into single cells with trypsin. Dissociated cells were transferred to a conical tube containing stop *medium* to neutralize trypsin. Cells were centrifuged for 5 min at 850 rpm, washed carefully two times with SFD medium and counted. For AFE induction, 25000-30000 cells/cm^2 were plated on fibronectin–coated (F0895, Sigma-Aldrich) 12-well tissue culture plates in AFE induction *medium* 1 [SFD medium supplemented with 10 mM SB-431542 (cat.#1614, Tocris) and 100 ng/mL of NOGGIN (cat.#6057, R&D Systems). After 24h of incubation, the *medium* was aspirated and AFE induction *medium* 2 [SFD medium suppplemented with 1 μM IWP2 (cat.#3533, Tocris) and 10 μM of SB-431542] was added to the cultures.

Lung progenitor induction and expansion

On day 6,5-7, AFE cultures treated for 20 days with the ventralization *medium* consisting of SFD *medium* supplemented with 3 μM CHIR99021 (cat.#04, Tocris), 10 ng/mL human FGF10 (cat.#345-FG, R&D Systems), 10 ng/mL human KGF (cat.#251-KG-010, R&D Systems), 10 ng/mL human BMP4 (cat.#314-BP, R&D Systems), 10 ng/mL murine EGF (cat.#2028-EG-200, R&D Systems) and 50 nM all-trans retinoic acid (cat.#R2625, Sigma-Aldrich). Culture *medium* was changed every two days. At a time point between days 8 to 12 cultures were incubated under normoxic conditions. At day 16, cultures were briefly digested with trypsin in order to remove potential non-ectodermal contaminating cells. Supernatant of this brief digestion containing single cells and small clumps were removed. The remaining cell clumps were replated onto fibronectin-coated MW12 plates at 1:3 dilutions in fresh *medium* after trypsin neutralization and careful washing. Plates were returned to the hypoxic conditions (5% CO_2/5%O_2/95%N_2 environment)

Lung and airway epithelial maturation

At day 26 cultures were incubated with SFD *medium* supplemented with 3 μM CHIR99021, 10 ng/mL human FGF10, 10 ng/mL human FGF10, 0,1 mM 8-bromo-cAMP (cat.# B5386, Sigma-Aldrich), 0,1 mM IBMX (3,7-dihydro-1-methyl-3-(2-methylpropyl)-1*H*-purine-2,6-dione; cat.# I5879, Sigma-Aldrich,)) and 60 nM dexamethasone (cat.#D5902, Sigma-Aldrich). The *medium* was changed every two days and plates were maintained under hypoxic conditions (5%CO_2/5%O_2/95%N_2 environment). Cultures were carried further in these conditions until their experimental use at day 106.

Assembly of minilungs on chamber slides

Glass chamber slides were treated for 3 hours at 37ºC with human fibronectin in order to be plated with terminally differenciated epithelial cells. Cultures from day 106 were digested with trypsin, neutralized with stop *medium* and washed with SFD *medium*. Approximately 30000 epithelial cells and 10000 primary myofibroblasts [human epithelial cells (Abyntek, Spain) that underwent spontaneous transition to

myofibroblasts] *per well* were plated in the epithelial maturation *medium* without dexamethasone. Cultures were maintaned in normoxic conditions for two days.

Viruses, purification and infection of minilungs

HEp-2 (ATCC) were maintained in DMEM medium supplemented with 10% FBS (F7524, Sigma-Aldrich), 2 mM glutamine (17605E, Lonza) and 100 U/ml of penicillin and streptomycin. The Long of HRSV was propagated in HEp-2 cells in medium supplemented with 2% FBS, glutamine and antibiotics. Viruses were purified and titrated in HEp-2 cells as previously described[2]. Assembled minilungs from day 108 were infected at a multiplicity of infection (moi) 1 or 0,1. The *inocula* (100 μL) consisted of purified virus diluted in maturation *medium* without dexametasone. After 90 min of virus adsorption, the *inocula* were removed and fresh medium was added (400 μL). At the indicated times the culture supernatants were collected for virus titration and the cultures were processed for immunofluorescence and RNA extraction.

Indirect Immunofluorescence

Cultures were fixed in 2% PFA in PBS for 10 min at RT and permeabilized with PBS-BSA 1%-0.25% Triton X-100 (5 min/RT). Preparations were then washed with PBS, blocked for 30 min with PBS/BSA1% and incubated overnight with antibodies against NKX2-1(1:100; ab76013, Abcam), SOX2 (1:100; C-17 sc-17320, Santa Cruz Biotech.), PDPN (1:100, sc-376695, Santa Cruz Biotech.), SFTPC (1:100, ab-211326, Abcam), ACTA2 (1:500; MA5-11547, Thermo Scientific), mix of antibodies against HRSV[3,4] (1:100), polyclonal Ab directed against the F glycoprotein of HRSV (476-510)[3]. Preparations were then washed with PBS and incubated with secondary antibodies conjugated with AF488, AF546 or AF555 (cats.#A11035 and A11001 (Life Technologies); cat.#150129, abcam) for 1h at RT. *Nuclei* were counterstained with DAPI, and samples were mounted with ProLong Diamond (cat.#P36961, Life Technologies). Cell images were captured with a fluorescence microscopy (Zeiss Axio) equipped with a camera (Axiocam MRm) and AxioVision software.

Quantitative real-time RT-PCR (qRT-PCR)

Total RNA was extracted using Trizol (cat.#15596026, Ambion) following manufacturer's instructions. cDNA was generated using the High-Capacity Reverse Transcription Kit from Quanta Biosciences (cat.#95047-025). Real-time qPCR was performed on a 7500HT FAST system (Applied Biosystems) using the 2X SYBR green reaction mix (cat.#10555100, Roche) following manufacturer's instructions. Absolute cuantification of each gene was obtained using a standard curve of serial diluted genomic DNA (cat.#11807720, Roche) and normalized to housekeeping gene TBP (Tata Box Binding protein). Primers sequences used were:

Gen (Hugo Genes)	Secuencia de los oligonucleótidos
TBP (TATA-BOX BINDING PROTEIN)	*Forward*: 5'-AGTTGCTCATACCGTGCTGCTA<br>*Reverse*: 5'- CCCTCAAACCAACTTGTCAACAGC
NKX2-1(NK2 HOMEOBOX 1)	*Forward*: 5'- CGGCATGAACATGAGCGGCAT<br>*Reverse*: 5'- GCCGACAGGTACTTCTGTTGCTTG
FOXA2	*Forward*: 5'- TTCAACCACCCGTTCTCCATCAAC

(FORKHEAD BOX A2)	*Reverse*: 5'- TGTTCGTAGGCCTTGAGGTCCATT
TP63 (TUMOR PROTEIN P63)	*Forward*: 5'- CCTATAACACAGACCACGCGCAGAA<br>*Reverse*: 5'- GTGATGGAGAGAGAGCATCGAAG
MUCIN5AC	*Forward*: 5'- GCACCAACGACAGGAAGGATGAG<br>*Reverse*: 5'- CACGTTCCAGAGCCGGACAT
SCGB1A1 (SECRETOGLOBIN FAMILY 1A MEMBER 1; CC10)	*Forward*: 5'- TCATGGACACACCCTCCAGTTATGAG<br>*Reverse*: 5'- TGAGCTTAATGATGCTTTCTCTGGGC
PDPN (PODOPLANIN)	*Forward*: 5'- AGGAGAGCAACAACTCAACGGGAA<br>*Reverse*: 5'- TTCTGCCAGGACCCAGAGC
(AQP5) AQUAPORIN 5	*Forward*: 5'- GCCATCCTTTACTTCTACCTGCTC<br>*Reverse*: 5'- GCTCATACGTGCCTTTGATGATGG
(SFTPA) SURFACTANT PROTEIN A	*Forward*: 5'- GTGCGAAGTGAAGGACGTTTGTGT<br>*Reverse*: 5'- TTTGAGACCATCTCTCCCGTCCC
SFTPB (SURFACTANT PROTEIN B)	*Forward*: 5'- TCTGAGTGCCACCTCTGCATGT<br>*Reverse*: 5'- TGGAGCATTGCCTGTGGTATGG
SFTPC (*SURFACTANT PROTEIN C)*	*Forward*: 5'- CCTTCTTATCGTGGTGGTGGTGGT<br>*Reverse*: 5'- TCTCCGTGTGTTTCTGGCTCATGT
SFTPD (*SURFACTANT PROTEIN D)*	*Forward*: 5'- TGACTGATTCCAAGACAGAGGGCA<br>*Reverse*: 5'- TCCACAAGCCCTGTCATTCCACTT

The analysis of gene expression induced by HRSV was performed by using TaqMan MGB probes (6-carboxyfluorescein [FAM] dye labeled) for the following genes: *ACTB* (Hs99999903_m1), *ISG15* (Hs00192713_m1), *DDX58* (Hs00204833_m1); *IL6* (Hs00985639_m1)*, CXCL8* (Hs00174103_m1), *TNF* (Hs01113624_g1). Applied Biosystems). Gene expression was normalized to the *ACTB* expression, and the comparative cycle threshold ($\Delta\Delta C_T$) method was used for relative quantifications.

Statistical analysis

Statistical significance of *data* was determined by applying a two-tailed Student's t test or analysis of variance followed by the Newman–Keuls or Bonferroni post-tests for experiments with more than two experimental groups. P <0.05 is considered significant. Significance of analysis of variance post-test or the Student's t test is indicated in the figures as *, P < 0.05; **, P <0.01; and ***, P < 0.001. Statistics were calculated with the Prism 7 software (GraphPad Software). The results presented in the figures are means ±SD.

Ethics statement

All experiments with the hESC line AND-1 were approved by the "Spanish National Commitee of Guarantees for the Donation and Use of Human Cells and Tissues"(ISCIII), approval number 345 288 1. Experimental protocols with animals were appproved by Commitee of Animal Welfare of the Community of Madrid (file reference: PROEX 312) following the guidelines for animal protection reported by the Spanish national law RD 53/2013.

1 Zambrano, A. *et al.* The thyroid hormone receptor beta induces DNA damage and premature senescence. *J Cell Biol* **204**, 129-146, doi:10.1083/jcb.201305084 (2014).
2 Martinez, I., Lombardia, L., Garcia-Barreno, B., Dominguez, O. & Melero, J. A. Distinct gene subsets are induced at different time points after human respiratory syncytial virus infection of A549 cells. *The Journal of general virology* **88**, 570-581, doi:10.1099/vir.0.82187-0 (2007).
3 Garcia-Barreno, B. *et al.* Marked differences in the antigenic structure of human respiratory syncytial virus F and G glycoproteins. *Journal of virology* **63**, 925-932 (1989).
4 Martinez, I., Dopazo, J. & Melero, J. A. Antigenic structure of the human respiratory syncytial virus G glycoprotein and relevance of hypermutation events for the generation of antigenic variants. *The Journal of general virology* **78 (Pt 10)**, 2419-2429 (1997).
